# Exercise training reduces workload, improves physical performance, and promotes overall health in welders

**DOI:** 10.1002/1348-9585.12122

**Published:** 2020-04-22

**Authors:** Christopher Weyh, Christian Pilat, Torsten Frech, Karsten Krüger, Thomas Reichel, Frank‐Christoph Mooren

**Affiliations:** ^1^ Department of Sports Exercise Physiology and Sports Therapy Justus‐Liebig‐University Giessen Germany; ^2^ Department of Rehabilitation Science Witten/Herdecke University Witten Germany

**Keywords:** endurance training, health promotion, manual handling tasks, musculoskeletal disorders, resistance training, welding

## Abstract

**Objectives:**

Welders demonstrate a significant prevalence of work‐related musculoskeletal disorders as indicated by high rates of illness‐related absenteeism. The aim of the study was to investigate the effects of a 24‐week exercise program on workload, physical performance, and overall health in welders.

**Methods:**

Seventy‐seven professional welders were assigned to either a control group (CG), an endurance training group (ETG), or a strength training group (STG). Both groups conducted a 24‐week, standardized and progressive endurance or resistance exercise training program. Before (TP1) and after training (TP2) all participants performed an experimental welding task (EWT) in order to test the hypothesis that training would reduce the relative load (%MVC) of eight skeletal muscles measured by surface electromyography. Secondary outcome measures included further EWT‐induced stress parameters and a series of health‐related outcome measures.

**Results:**

Results revealed a lower muscle load in participants of the ETG and STG for trapezius muscle at TP2 compared to T1 (*P* < .05 vs CG). Rate of perceived exertion and visual analogue scale were decreased, while increase of maximum EWT duration was found in participants of the ETG and STG after training (*P* < .05 vs CG). At T2, body fat (%) decreased and physical performance (bicycle exercise test, isometric strength of core muscles) increased in ETG and STG (*P* < .05).

**Conclusion:**

Both regular endurance and strength training represent effective strategies for reducing workload and improving physical performance of welders. The results emphasize the importance of physical fitness for welders and might motivate health professionals in steel‐industry to offer access to exercise training programs.

## 1 INTRODUCTION

Musculoskeletal disorders are one of the most prominent causes of functional disability and pain in industrialized world and consume a large amount of health and social resources.^1^ Work‐related musculoskeletal disorders (WMSD) resulting from work‐related strains can be considered as a major contributing factor to sick leave.^2^

The prevalence of WMSDs such as back pain and joint disorders are most common in occupations which are engaged in manual handling tasks and in professions with heavy physical demands.^3^ One occupational group with significant prevalence of WMSD among industrial workers are welders as indicated by high rates of illness‐related absenteeism and early invalidity.^4^, ^5^, ^6^ This is not surprising since welders perform most of their work in extended forced postures (EFP).^7^, ^8^ EFP are frequently required in many welding applications characterized in bended or overhead positions. They cause a reduced peripheral blood flow and premature muscle fatigue^9^ resulting in malposition's which ultimately lead to an awkward force transmission and hereby may hazard joints, ligaments, and bursae.^10^ Overall, EFP exert unphysiological loads on the musculoskeletal system and could compromise musculoskeletal health in the long‐term.

There is evidence that physical training programs can have positive effect on physical capacity of workers in general.^11^, ^12^ Numerous studies demonstrated the effects of strength training in the prevention of lower back and neck pain.^13^, ^14^, ^15^, ^16^, ^17^, ^18^ Recently, our group evaluated the effect of a resistance training program.^19^ In this pilot study, a 12‐week resistance training program was able to reduce muscle stress response during welding task in novices. However, these results need to be confirmed in professional welders to derive specific training recommendations in terms of time, duration, and modality of exercise. To date, there are no studies available comparing resistance and endurance training programs in the long term and under everyday conditions in professional welders. Therefore, the objective of the present study was to investigate the effects of an individualized, progressive 24‐week endurance or strength training program on workload, physical performance, and overall health, by means of both objective bio‐medical and subjective psycho‐social parameters, in professional welders.

## 2 PARTICIPANTS AND METHODS

### 2.1 Study design

This study was conducted as an interventional study with a pre‐post‐test design. Since a randomization to the intervention groups was not accepted by some of the participants the investigators decided to adapt the design to a quasi‐experiment where the participants choose their groups themselves. Group 1 performed a 24‐week strength training (strength training group [STG]) which was specially designed for the demands during welding. Group 2 performed a 24‐week endurance training (endurance training group [ETG]) and Group 3 received no additional treatment (control group [CG]). All measurements were conducted before training (T1) and after training (T2). As main outcome measure, the relative load of trapezius muscle was chosen.

### 2.2 Participants

Seventy‐seven participants (76 male; 1 female [ETG]) completed the 24‐week intervention and were included into data analysis. Their age and anthropometrics are shown in Table 1. Participants were recruited by calls in newspaper, mail or by telephone, as well as together with employers. Inclusion criteria were age ≥20 and ≤59 years, welding experience of at least 3 months, welding time at least 4 hours per day, and a low frequency of physical activity (<6 hours per week). Exclusion criteria were acute coronary heart disease, severe bronchial asthma, poorly controlled diabetes, acute inflammatory, or febrile diseases, and generally any clinical condition that is a contraindication to exercising. This study was approved by the local ethics committee of the Justus‐Liebig‐University Giessen. All participants gave written informed consent before enrolment.

**TABLE 1:** Anthropometry of control group (CG), endurance training group (ETG), and strength training group (STG) (mean ± SD) and *P* value of analysis of differences between groups

	CG (n = 21)	ETG (n = 28)	STG (n = 28)	*P* value
Male (n)	21	27	28	
Age (y)	39 ± 11	39 ± 10	42 ± 8	.34
Height (cm)	176.4 ± 8.5	177.4 ± 7.7	177.4 ± 6.7	.88
Weight (kg)	87.8 ± 17.1	92.0 ± 20.7	87.6 ± 12.0	.57
BMI (kg/m^2^)	28.2 ± 4.5	29.3 ± 6.5	27.9 ± 3.5	.56

### 2.3 Experimental welding task

**FIGURE 1:**
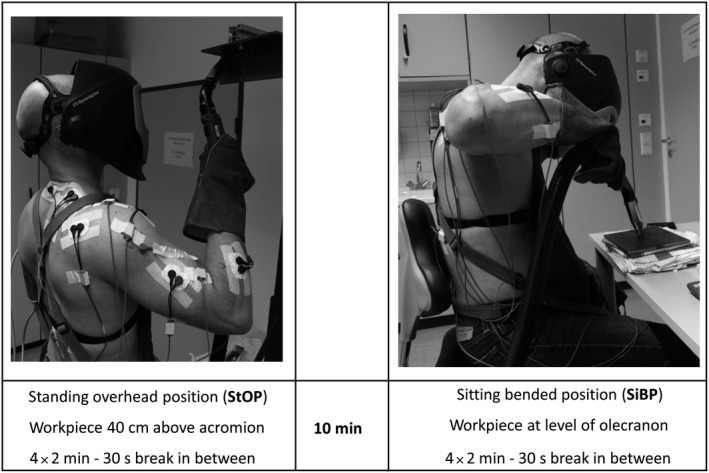
Experimental setup of the experimental welding task (EWT)

The EWT was designed to simulate the occupational workload of welders by means of a standardized laboratory experiment and was performed without electricity and no welding light to avoid interferences with the surface electromyography (SEMG) and to increase work safety, respectively. The EWT was performed in two positions: First, the participants performed EWT in a standing overhead Position (StOP) (ASME: 4F) (Figure 1). The position of the workpiece was adjusted exactly 40 cm above the individual acromion height. Second, the participants had to weld in sitting bended position (SiBP) (ASME: 1G) (Figure 1). Here, the participants sat on a chair and the workpiece was positioned at the height of the olecranon (90° flexed position of the elbow). The rationale for these positions was that they are common from EFP in industrial workers and exert a maximum of physical effort as shown by our own group and others.^19^, ^20^ The task was to weld a fictitious seam of two metal‐workpieces. In both positions, all participants welded one‐handed with their dominant hand. The welding speed was standardized by a metronome (20 beats/min). Working angle, travel angle, and contact‐to‐work‐distance were standardized as far as possible, too. The participants were encouraged to simulate the real conditions as much as possible. Both EWT positions were performed 4 times for 120 seconds (sec) with a rest of 30 seconds between the passes and a rest of 10 minutes between both positions. The EWT was carried out with a real welding torch weighing 3.6 kg (RAB Plus 36kD®, Alexander Binzel Schweisstechnik GmbH & Co. KG).

### 2.4 Surface electromyography

Surface electromyography (SEMG) was used to determine the relative muscle activity load throughout the EWT. SEMG signals were determined from shoulder‐neck (deltoideus pars anterior m., trapezius pars descendens m., infraspinatus m., pectoralis major pars clavicularis m.), core (erector spinae m. at the height of lumbar vertebrae 1), and arm muscles (triceps brachii m., biceps brachii m., extensor digitorum longus m.) by a bipolar wireless eight channel system (Noraxon^®^ Inc USA). Preparation and electrode (single self‐adhesive sensor Ag/AgCl‐electrodes, Co‐med^®^, GolTec) placement (middle of the muscle belly with 2 cm interelectrode distance) were consistent with the SENIAM guidelines for SEMG recordings.^21^ A ground electrode was placed on cervical vertebrae 7. Before applying the electrodes, the skin was shaved, scrubbed, and cleaned with alcohol. After electrode placement, the skin resistance was checked to be less than 10 kΩ. SEMG signals were recorded and stored on a portable PC during the welding activity. Raw signals were amplified 1000 times over a frequency range of 5‐1000 Hz, digitized with an A/D converter at 5 kHz and sampled at 1500 Hz. The signal processing consisted of full wave rectification and smoothing using a root mean square algorithm with a 100 ms time constant. Ultimately, the signals were controlled visually for the presence of artefacts or noise and to see if artefacts were cleared through adequate filters. SEMG data were normalized of the basis of electrical activity during a maximal voluntary contraction (MVC) of each muscle which were determined in a standardized pre‐test. For this purpose, the ratio of the mean amplitude during each EWT trial and the mean amplitude of the highest 500 ms activity period during an MVC exercise was defined as indicator of the relative workload of the individual muscle (%MVC).

### 2.5 Cardiovascular and subjective exertion

During EWT heart rate was analyzed using a Polar® Heart Rate Monitor RS800G3 (Polar® Electro GmbH Büttelborn). Maximum heart rate (HRmax) was calculated at the end of all four passes. Maximum blood pressure (systolic [SBPmax]/diastolic [DBPmax]) was measured using an electronic blood pressure monitor (BOSO® TM‐2430 PC, Bosch and Sohn) at the end of all four passes, too. Immediately after finishing each EWT position, the study participants rated their maximum perceived pain upward from the hips and maximum overall exertion by means of a 100‐mm visual analogue scale (VASmax) and Borg rating of perceived exertion scale (6‐20) (RPEmax), respectively.

### 2.6 Physical performance and overall health parameters

Body fat (%) and muscle mass (%) were analyzed by bioelectric impedance analysis (BIA) (Model 101 Anniversary Sport Edition, Akern Srl®).

A bicycle exercise test (initial workload 50 Watt, increased each 2 minutes by 25 Watt) was performed until exhaustion (electrical braked cycle ergometer—Excalibur Sport®, Lode). Therefore, we measured maximum bicycle performance (maximum workload in watt [W]) and relative bicycle performance in relation to body weight (BW) (W/kg/BW) as outcome measures to quantify endurance capacity. Maximum bicycle performance (W) was calculated according to the formula: workload last stage completed (W) + [time of the last uncompleted stage (sec)/stage duration (sec) × stage increments (W)].

Maximum strength tests were performed to measure muscular capacity. Peak isometric torque (Newton meter [Nm]) during elbow flexion and extension, knee flexion and extension, as well as trunk flexion and back extension were measured by the m3‐Diagnos analysis station (Schnell). Two trials were completed for each position, with contractions lasting 5 seconds, separated by 30 seconds rest intervals. Participants were encouraged verbally to elicit their maximal effort (and force was displayed on a visual display in real time providing immediate feedback). Peak torque values (Nm) were recorded, and the higher of the two repetitions was used for statistical analysis.

In addition, health‐related quality of life was assessed by the SF‐36 questionnaire. Physical component summary (PCS) and mental component summary (MCS) were calculated.

### 2.7 Exercise training program

The training program followed exercise guidelines for the general population from the American College of Sports Medicine.^12^, ^22^, ^23^

**FIGURE 2:**
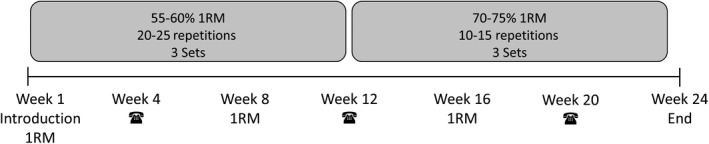
Procedure and training parameters for strength training program. 1RM One repetition maximum, ☎ Telephone call by the instructor to increase motivation and compliance of the participants

Participants of the STG performed a whole‐body strength training program and included a periodized variation of program variables. Precisely, during the first 12 weeks, participants performed the training in 3 sets of 20‐25 repetitions at 55%‐60% of their one repetition maximum (1RM). At the second 12 weeks, training was performed in 3 sets of with 10‐15 repetitions at an intensity of 70%‐75% of 1RM. There was a 60‐sec break between each set. The resistance training program considered those muscles which are stressed during welding most such as back, shoulder‐neck, and forearm and their antagonists, too. All participants received an introduction by a sports scientist and performed the strength training 2‐3 times a week self‐depended thereafter. The exercise intensity was adapted to the individual training process as shown by additional measurements of the 1RM at weeks 8 and 16. To increase the compliance the investigator contacted the study participants by telephone at week 4, 12, and 20 (Figure 2). Each session started with a global warming up of 10 minutes. The training program consisted of the following exercises: Pectoralis muscle was trained by using chest or bench press, deltoideus muscle was trained by (dumbbell) shoulder raises. Upper back exercise included seated row (rhomboidei mm.) and dumbbell neck lift (trapezius muscle). Flexors and extensors palmaris mm. were trained by forearm dumbbell curls. Muscles of rotator cuff were trained by cable internal (supraspinatus muscle) and external rotation (infraspinatus muscle). Core exercise included back extension (erector spinae muscle) abdominal crunch on device or common crunches (rectus abdominis muscle). Based on whole body training, leg press (quadriceps femoris muscle and ischiocrucrales muscle) was also included into the exercise program. As the participants were employed resident all over Germany, the training facility were chosen according to the following characteristics: nearby work or home and possibility to perform the prescribe exercises.

**FIGURE 3:**
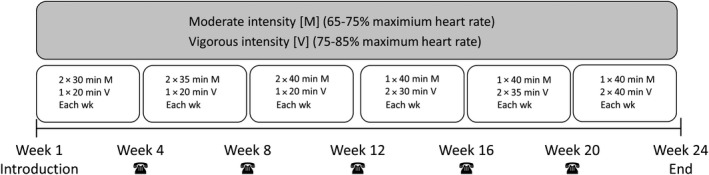
Procedure and training parameters for endurance training program. 1RM One repetition maximum, ☎ Telephone call by the instructor to increase motivation and compliance of the participants

Participants of the ETG either performed cycling, jogging, or (nordic‐) walking according to their own preferences thrice a week separated by a minimum of 24 hours rest. Endurance training program was designed in a standardized and progressively way and included a periodized variation of program variables, too. In weeks 1‐12, participants performed moderate intensity twice and vigorous intensity once. Duration of moderate intensity increased from 30 minutes each 4 weeks by 5 minutes up to 40 minutes, while vigorous intensity stayed consequently at 20 minutes. At the beginning of week 13 up to week 24, participants performed moderate intensity once and vigorous intensity twice. Duration of vigorous intensity increased from 30 minutes each 4 weeks by 5 minutes up to 40 minutes, while moderate intensity keeps consequently at 40 minutes. Therefore, training volume increased in both periods every 4 weeks by 10%. Moderate intensity was defined as 65%‐75% of individual maximum heart rate and a vigorous intensity was defined as 75%‐85% of individual maximum heart rate. The highest recorded heart rate at the end of the final stage of the bicycle exercise test was regarded as maximum heart rate. The participants controlled their training intensity themselves by wearing a heart rate monitor Polar® Heart Rate Monitor FT1 (Polar® Electro GmbH Büttelborn). In weeks 4, 8, 12, 6, and 20, the participants were contacted by telephone in addition to the training protocol (Figure 3). Participants of CG received no treatment.

The training programs were completed self‐dependent during leisure‐time. Each participant got a written training plan and was asked to keep as closely as possible to the program. All completed and missed training sessions, for example, due to sick leave or exceptional events, as well as any deviations from the prescribed program had to be documented by themselves. The number of self‐reported accomplished training sessions were averaged and termed “attendance rate.”

### 2.8 Statistical analysis

The results were analyzed according to the intention‐to‐treat principle. All participants who attended to the post‐test were included for data analysis. Statistical analysis was performed by SPSS version 24 (IBM® SPSS Statistics 24, IBM GmbH). As all data were normally distributed (Kolmogorov‐Smirnov test), parametric tests could be applied for further analysis. Initial baseline measurements were analyzed by analysis of variance (ANOVA) to determine differences between the groups. To determine the interaction of group x time point and time effect a two‐way ANOVA with repeated measures (3 × 2) was calculated. Bonferroni‐Holm corrected tests were used for post hoc analysis to detect differences between groups and time points. Due to the explorative nature of the investigation of all secondary outcome parameters, no adjustment of the alpha error for multiple testing has been considered. If not indicated otherwise, results are given as arithmetic mean ± standard deviation (SD). The level of statistical significance was set at *P* ≤ .05.

## 3 RESULTS

In total, attendance rate of ETG amounted to 77% ± 25% (55 ± 18 of 72 training sessions) and attendance rate of STG amounted to 66% ± 21% (40 ± 13 of 60 training sessions).

### 3.1 Relative muscle load (%MVC)

**FIGURE 4:**
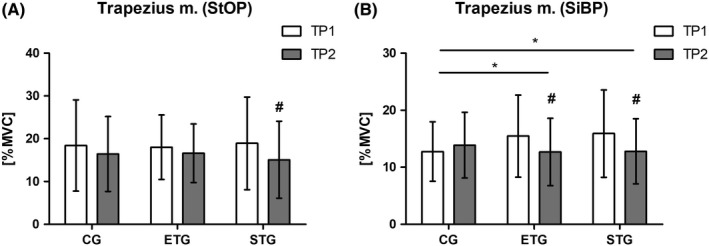
Relative muscle load (%MVC) of main outcome parameter trapezius m. Control group (CG), endurance training group (ETG), and strength training group (STG) standing overhead position (StOP) (A) and sitting bended position (SiBP) (B). ^#^ *P* ≤ .05 post hoc differences from TP1 to TP2 within groups, *P ≤ .05 post hoc differences between groups

The two‐way repeated‐measure ANOVA revealed a significant effect for time (*F*_1,73_ = 5.93; *P* = .017) but no time × group (*F*_2,73_ = 0.61; *P* = .547) in standing overhead position (StOP). Post hoc analysis showed significant reduction of muscle load between TP1 and TP2 in STG (18.9 ± 10.8‐15.1 ± 9.0%MVC; *P* = .048) (Figure 4A) but not when compared to CG (*P* = .30) or ETG (*P* = .30). The two‐way repeated‐measure ANOVA revealed a significant effect for time (*F*_1,71_ = 5.00; *P* = .028) and time × group (*F*_2,71_ = 3.63; *P* = .032) in sitting bended position (SiBP). Post hoc analysis of time effect showed significant reduction of muscle load between TP1 and TP2 in ETG (15.5 ± 7.2‐12.7 ± 6.0; *P* = .024) and STG (15.9 ± 7.7‐12.8 ± 5.7; *P* = .028) (Figure 4B). There were significant differences between ETG and CG (*P* = .045), as well as STG and CG (*P* = .048). A significant time effect for infraspinatus muscle in StOP was observed, while post hoc analysis showed no significant difference (*P* = .078). In SiBP, analysis of the erector spinae muscle revealed a significant effect for time × group. Post hoc analysis revealed no significant differences between groups, too (ETG vs CG: *P* = .081; STG vs CG: *P* = .086). A significant time and time × group effect were detected for pectoralis major muscle in SiBP. The post hoc analysis showed a significant difference within STG (*P* = .012) and between STG vs CG (*P* = .003) (Table 2).

**TABLE 2:** Relative muscle load (%MVC) at TP1 and TP2 in control group (CG), endurance training group (ETG), and strength training group (STG) in standing overhead position (StOP) and sitting bended position (SiBP) (means ± SD)

	CG		ETG		STG		Time effect	Time × group interaction
	TP1	TP2	TP1	TP2	TP1	TP2		
StOP								
Erector spinae m.	6.7 ± 5.2	6.7 ± 4.1	6.1 ± 4.4	5.6 ± 2.9	5.7 ± 4.2	4.9 ± 2.5	n.s.	n.s.
Infraspinatus m.	12.0 ± 9.7	10.6 ± 6.1	12.4 ± 4.9	11.7 ± 5.7	11.3 ± 7.3	8.4 ± 5.2	0.031	n.s.
Deltoideus m.	16.5 ± 6.2	17.2 ± 7.2	17.5 ± 7.8	17.2 ± 7.2	14.5 ± 4.6	12.0 ± 6.2	n.s.	n.s.
Pectoralis major m.	12.0 ± 6.4	11.8 ± 6.4	11.4 ± 6.8	11.9 ± 7.9	9.9 ± 5.2	12,0 ± 7.1	n.s	n.s.
Extensor dig. long m.	16.2 ± 8.0	14.6 ± 6.8	14.3 ± 4.5	16.7 ± 6.6	14.5 ± 6.2	14.7 ± 5.5	n.s.	n.s.
Biceps b. m.	3.3 ± 2.9	4.1 ± 3.3	2.6 ± 1.4	3.2 ± 2.3	4.7 ± 3.8	2.9 ± 2.5	n.s.	n.s.
Triceps b. m.	4.2 ± 3.1	8.0 ± 9.2	2.7 ± 1.6	3.1 ± 3.7	5.7 ± 3.9	4.4 ± 4.7	n.s.	n.s.
SiBP								
Erector spinae m.	4.6 ± 3.0	6.8 ± 4.9	7.9 ± 5.6	6.4 ± 3.9	6.9 ± 4.7	5.9 ± 3.9	n.s.	0.023
Infraspinatus m.	9.8 ± 6.2	10.5 ± 5.9	11.7 ± 4.7	9.3 ± 4.3	9.7 ± 5.4	9.4 ± 7.0	n.s.	n.s.
Deltoideus m.	9.5 ± 6.2	8.9 ± 4.0	8.3 ± 5.1	6.5 ± 3.9	7.6 ± 6.4	6.2 ± 4.0	n.s.	n.s.
Pectoralis major m.	2.7 ± 2.2	2.2 ± 1.9	2.5 ± 3.5	3.1 ± 5.8	2.5 ± 2.4	6.1 ± 6.1	0.030^a^	0.008^b^
Extensor dig. long m.	8.9 ± 4.8	10.0 ± 7.2	6.8 ± 7.1	8.6 ± 5.4	9.8 ± 5.2	9.6 ± 6.0	n.s.	n.s.
Biceps b. m.	3.7 ± 2.5	4.2 ± 3.4	5.3 ± 5.0	4.2 ± 2.9	6.0 ± 4.9	4.2 ± 3.2	n.s.	n.s.
Triceps b. m.	4.9 ± 3.5	8.9 ± 8.6	3.8 ± 3.8	3.5 ± 4.3	7.6 ± 4.3	6.5 ± 5.9	n.s.	n.s.

Abbreviation: n.s., not significant.

^a^ *P* ≤ .05 post hoc differences from TP1 to TP2 within STG.

^b^ *P* ≤ .05 post hoc differences between CG and STG.

### 3.2 Heart rate, subjective exertion, and EWT‐duration

A significant time effect for HRmax was detected in StOP (ETG: *P* = .018) and in SiBP (ETG: *P* = .030). The analysis of subjective exertion showed a significant time effect for RPEmax in StOP (ETG: *P* ≤ .001; STG: *P* = .012) and SiBP (ETG: *P* ≤ .001). Additionally, there was a time × group effect for RPEmax in StOP (ETG vs CG: *P* = .009; STG vs CG: *P* = .040) and SiBP (ETG vs CG: *P* = .006). Furthermore, we detected a significant time effect for VASmax in StOP (STG: *P* = .012). Data analysis revealed further a significant time × group effect in StOP while there were no significant difference in post hoc analysis between groups. However, in SiBP there was a significant difference between ETG vs CG (*P* = .012) and STG vs CG (*P* = .015). Additionally, we found a significant time effect for EWT‐duration (ETG: *P* = .012; STG: *P* = .006) and a time × group effect (ETG vs CG: *P* = .05, STG vs CG: *P* = .024) in StOP (Table 3).

**TABLE 3:** Cardiovascular and subjective parameters at time point 1 (TP1) and TP2 in control group (CG) endurance training group (ETG) and strength training group (STG) in standing overhead position (StOP) and sitting bended position (SiBP) (means ± SD)

	CG		ETG		STG		Time effect	Time × group interaction
	TP1	TP2	TP1	TP2	TP1	TP2		
StOP								
SBPmax (mm Hg)	156 ± 27	157 ± 24	154 ± 16	158 ± 18	152 ± 24	150 ± 17	n.s.	n.s.
DBPmax (mm Hg)	107 ± 13	105 ± 10	109 ± 13	105 ± 10	103 ± 12	103 ± 10	n.s.	n.s.
HRmax (beats/min)	99 ± 14	95 ± 15	98 ± 16	91 ± 11	100 ± 16	95 ± 14	0.002^a^	n.s.
RPEmax (Borg)	16 ± 3	16 ± 3	16 ± 2	15 ± 2	16 ± 2	15 ± 2	0.003^a^, ^b^	0.009^c^, ^d^
VASmax (mm)	48 ± 30	52 ± 28	48 ± 23	40 ± 25	50 ± 29	34 ± 27	0.042^b^	0.048
EWT‐duration (s)	428 ± 77	428 ± 79	439 ± 62	468 ± 31	424 ± 67	458 ± 45	≤0.001^a^, ^b^	0.021^c^, ^d^
SiBP								
SBPmax (mm Hg)	155 ± 24	152 ± 23	151 ± 19	143 ± 13	150 ± 23	150 ± 23	n.s.	n.s.
DBPmax (mm Hg)	107 ± 14	103 ± 12	103 ± 11	97 ± 10	100 ± 12	102 ± 18	n.s.	n.s.
HRmax (beats/min)	87 ± 11	84 ± 10	87 ± 16	80 ± 15	89 ± 15	84 ± 12	≤0.001^a^	n.s.
RPEmax (Borg)	14 ± 4	15 ± 3	15 ± 1	13 ± 2	15 ± 2	14 ± 2	≤0.001^a^	0.004^c^
VASmax (mm)	39 ± 29	47 ± 26	37 ± 21	26 ± 17	41 ± 24	30 ± 25	n.s.	0.008^c^, ^d^
EWT‐duration (s)	463 ± 47	463 ± 47	464 ± 50	476 ± 20	471 ± 33	478 ± 11	n.s.	n.s.

Abbreviations: DBPmax, maximum diastolic blood pressure; EWT, experimental welding task; HRmax, maximum heart rate; n.s., not significant, RPEmax, maximum rating of perceived exertion; SBPmax, maximum systolic blood pressure; VASmax, maximum visual analogue scale.

^a^ *P* ≤ .05 post hoc differences from TP1 to TP2 within ETG.

^b^ *P* ≤ .05 post hoc differences from TP1 to TP2 within STG.

^c^ *P* ≤ .05 post hoc differences between CG and ETG.

^d^ *P* ≤ .05 post hoc differences between CG and STG.

### 3.3 Body composition, physical performance, and health‐related quality of life

Body fat (%) revealed a significant time effect (ETG: *P* = .042; STG: *P* = .006) and time × group effect (ETG vs CG: *P* = .042; STG vs CG: *P* = .006). Muscle mass (%) showed a time effect, too (ETG: *P* = .050; STG: *P* = .003), but no interaction. Among the bicycle exercise test a significant time effect in maximum bicycle performance (W) (ETG: *P* ≤ .001; STG: *P* = .024), as well as relative bicycle performance (W/kg/BW) (ETG: *P* = .003) was found. Moreover, we detected significantly time × group effects for maximum bicycle exercise (W) (ETG vs CG: *P* = .009; STG vs CG: *P* = .016) and relative bicycle exercise (W/kg/BW) (ETG vs CG *P* = .027). The results of maximum strength test revealed a significant time x group effect of trunk flexion (ETG vs CG: *P* = .008; STG vs CG: *P* ≤ .001). Additionally, there was a significant time effect (ETG: *P* = .030; STG: *P* ≤ .001) and time × group effect (ETG vs CG: *P* = .046; STG vs GC: *P* = .003) for back extension (Table 4).

**TABLE 4:** Body composition, physical performance, and health‐related quality of life at time point 1 (TP1) and TP2 in control group (CG) endurance training group (ETG) and strength training group (STG) (means ± SD)

	CG		ETG		STG		Time effect	Time × group interaction
	TP1	TP2	TP1	TP2	TP1	TP2		
Weight (kg)	87.8 ± 17.1	88.2 ± 18.8	92.0 ± 20.7	90.8 ± 20.0	87.7 ± 12.0	88.2 ± 11.7	n.s.	n.s.
BMI (kg/m^2^)	28.2 ± 4.5	28.2 ± 4.5	29.3 ± 6.5	28.6 ± 6.2	27.9 ± 3.5	28.0 ± 3.5	n.s.	n.s.
Fat mass (%)	23 ± 6	24 ± 6	25 ± 7	23 ± 6	23 ± 6	21 ± 5	0.005^a^, ^b^	0.005^c^, ^d^
Muscle mass (%)	54 ± 6	54 ± 5	54 ± 5	55 ± 5	54 ± 4	57 ± 4	0.002^a^, ^b^	n.s.
Maximum bicycle performance (W)	202 ± 47	199 ± 43	206 ± 34	226 ± 37	207 ± 53	225 ± 45	≤0.001^a^, ^b^	0.006^c^, ^d^
Relative bicycle performance (W/kg/BW)	2.4 ± 0.5	2.4 ± 0.5	2.3 ± 0.6	2.5 ± 0.6	2.5 ± 0.7	2.7 ± 0.6	≤0.001^a^	0.036^c^
Arm flexion (Nm)	129.1 ± 23.4	131.5 ± 18.1	132.9 ± 34.0	138.1 ± 33.3	131.1 ± 24.9	135.9 ± 28.5	n.s.	n.s.
Arm extension (Nm)	71.0 ± 23.4	67.8 ± 19.6	70.5 ± 17.5	74.7 ± 21.6	75.1 ± 20.9	77.3 ± 19.1	n.s.	n.s.
Knee flexion (Nm)	206.5 ± 59.8	208.1 ± 45.9	201.4 ± 63.1	216.5 ± 71.3	188.4 ± 49.8	202.3 ± 53.7	n.s.	n.s.
Knee extension (Nm)	409.6 ± 120.8	394.4 ± 122.6	371.1 ± 111.6	399.5 ± 108.2	390.1 ± 92.8	416.2 ± 120.7	n.s.	n.s.
Trunk flexion (Nm)	181.1 ± 70.9	162.3 ± 56.0	151.8 ± 50.6	167.1 ± 57.9	143.1 ± 44.8	169.1 ± 60.5	n.s.	≤0.001^c^, ^d^
Back extension (Nm)	337.0 ± 126.9	303.0 ± 132.6	259.2 ± 109.4	287.1 ± 91.8	299.7 ± 93.0	373.2 ± 111.9	0.030^a^, ^b^	≤0.001^c^, ^d^
PCS (SF‐36‐score)	50.8 ± 6.0	49.7 ± 7.3	52.7 ± 4.8	52.4 ± 4.4	45.7 ± 7.8	52.0 ± 4.8	—	—
MCS (SF‐36‐score)	51.4 ± 7.4	50.1 ± 9.2	52.3 ± 4.0	53.2 ± 5.5	50.6 ± 8.6	53.2 ± 5.2	n.s.	n.s.

Abbreviations: BW, body weight; MCS, mental component summary; n.s., not significant; Nm, newton meter; PCS, physical component summary, W, watt.

^a^ *P* ≤ .05 post hoc differences from TP1 to TP2 within ETG.

^b^ *P* ≤ .05 post hoc differences from TP1 to TP2 within STG.

^c^ *P* ≤ .05 post hoc differences between CG and ETG.

^d^ *P* ≤ .05 post hoc differences between CG and STG.

Due to significant group differences for PCS at baseline no ANOVA was performed.

## 4 DISCUSSION

This study indicates that both a 24‐week strength as well as endurance training exert beneficial effects on work‐related stress and on different dimensions of health and performance in professional welders. Both exercise training interventions were effective in reducing the muscle workload, the degree of subjective exertion and the perceived pain intensity after EWT. In parallel, the maximum possible duration of the welding task increased when compared to control. Within ETG maximum heart rate decreased during welding task. Results slightly differed between StOP and SiBP relating to physical response. Strength and exercise capacity increased and body fat mass decreased following both interventions compared to control. Body muscle mass increased after strength and endurance exercise program. Overall health and performance parameter were improved equally by both interventions.

Regarding the MVC measures relative load of the trapezius muscle was chosen as primary outcome measure. Former studies demonstrated that the upper trapezius muscle indicated one of the highest stress during welding especially in overhead positions.^4^, ^19^, ^20^ This muscle plays a crucial role in the development of shoulder neck disorders.^24^ The results of the trapezius m. demonstrate a reduced relative muscular load after both training interventions. Especially the strength training program seems to be more favorable among both positions. Since the relative muscle load is defined as the ratio of SEMG activity during a given task and a maximum voluntary contraction (MVC) it can be assumed that the decrease in muscular workload is associated with increased muscular capacity. An increase of muscular capacity after strength training was expectable. However, there are only a few comparable studies with occupations engaged in manual handling tasks. These data provide heterogeneous results. While Hamberg‐van Reenen and colleagues^25^ could not show a reduction of muscular load of the trapezius muscle or other muscles during a manual assembly task after an 8‐week strength training intervention, a study of Krüger and colleagues^19^ showed a reduction in trapezius muscle load during a welding simulation after 12‐week of strength training. Besides a longer training period, a higher volume of sets (3 vs 2) may explain the different results, too.

Surprisingly, an increase in relative muscle load of pectoralis major m. in SiBP was found. Albeit the pectoralis m. showed the lowest load of all measured muscles during EWT. This muscle is an important synergist of stabilizing muscle groups of the shoulder, such as rotator cuff, especially of the subscapular and deltoid muscles.^26^ It is speculated that the strength training program induces changes of the muscle activation pattern observed during welding, hence activating structures with low stress (pectoralis m.) and relieving stressful structures (trapezius m.). This assumption is supported by Kadefors and colleagues^20^ who already showed differences in muscular activation profile between experienced and inexperienced welders. Similarly, a study among patients handlers demonstrated differences in muscle activation patterns between experienced and novices and proposed that this is a beneficial learning effect which helps to protect the spine.^27^ Therefore, it is speculated that the increased relative muscle load of the pectoralis m. may be a part of an altered muscle activation pattern and classified as a functional adaptation in order to protect structures of shoulder and upper neck.

Interestingly, the reduced muscular load of the trapezius muscle in SiBP was also detected in ETG. It is suggested that this is a result of ameliorated physical conditions as indicated by maximum bicycle and core strength test results and reflect improved welding ergonomics in general. We further assume that the alterations in relative muscle load may result in general health benefits and contribute to a reduced risk for shoulder‐neck disorders among welders in long‐term.

Altered muscle activation went along within a reduction of RPEmax and VASmax. This is not surprising, since a lower level of muscular load induces less physical exertion and muscular pain. VAS seems to be a good indicator of musculoskeletal symptoms, and also RPE is commonly used to evaluate job related strains.^28^, ^29^, ^30^ RPE is related to kinesthetic sensitivity, ligament, joint, tendon, and muscle proprioception and predicts perceived exertions better at lower levels of occupational activities in opposite to aerobic and systemic exercise where heart rate and blood lactate are better predictors.^5^ Indeed, a decreased response of HRmax during EWT and an increased endurance capacity were observed in the ETG which are common adaptive responses after a regular endurance training.^31^, ^32^ However, in the current study, the EWT did not induce a high cardiovascular response in general. Therefore, it is speculated that the decrease of VASmax and RPEmax was primarily associated with the reduction of muscular load.

The various effects of a regular resistance or endurance training on physical functions and health are well evaluated. Accordingly, the loss of body fat and increases in muscle mass are known reducing factors for cardiovascular diseases. Similarly, increased endurance capacity and muscular strength represents an important factor for overall health.^16^, ^33^, ^34^, ^35^, ^36^

Beside a positive effect on welders' health and wellbeing, companies are interested in a high productivity of their staff. In order to quantify working performance, EWT duration was analyzed, too. The present results show that a longer EWT duration could be achieved after both exercise programs in StOP. Consequently, we cannot exclude a bias on other outcome measures as a longer welding duration could result in a higher degree of fatigue.

Overall, the attendance rate to the training program was only moderate to poor, especially in STG. Albeit, we did not evaluate the reason for missing training sessions. From the overall feedback, we conclude that training program itself represent no barriers for attendance. Nevertheless, even two to three sessions of endurance training or one to two sessions of strength training per week seems to reduce workload, improves physical performance, and promotes overall health in welders. However, given a low attendance rate in this study, the authors would expect that a higher exercise frequency would be more effective which is in line with other studies.^37^ A higher adherence to exercise could be improved by workplace‐based training programs.^37^, ^38^ The present approach might be applicable to the majority of industry companies where no training opportunities exist those.

Anyway, this study has some limitations. First, a randomization was not applied. Consequently, a selection bias may have occurred in terms of unbalanced training experience, prevalence of MSD, and work experience over the study groups. For instance, it cannot be excluded that participants chose to take part in a training group due to their own training experience. If this were the case, training adaptation effects would be underestimated since untrained individuals have higher adaptation potentials. Furthermore, the EWT was performed under standardized laboratory conditions increasing the internal validity of this study. Anyhow, the external validity was negatively affected since welders are generally exposed to additional stressors such as weight of protective clothing, fumes, and heat but we could not imitate these extreme conditions for technical reasons. Last, information about both, attendance rate and adherence to training were derived from the participant's self‐report, which could bias the results.

In conclusion, this study demonstrated that a regular strength or endurance training induces various objective and subjective adaptations in welders. These data emphasize the benefit of a physical training in the prevention and health promotion of welders in terms of reducing workload, increasing physical performance, and improving overall health. Regarding our main outcome measures both exercise programs seem to have similar benefits, whereby strength training seems to be a bit more favorable. Secondary measures indicated differentiated effects for each exercise type without pointing out a superiority of one exercise type. While strength training has a greater impact on the muscular activation pattern endurance training exerts positive cardiovascular effects during welding. Both interventions lead to a decrease of subjective exertion. If these programs are also able to reduce WMSD, long‐term illnesses and painful conditions, and prevent work‐related absenteeism has to be evaluated in future studies.

## DISCLOSURES

*Approval of the research protocol*: This study was approved by the local ethics committee of the Justus‐Liebig‐University Giessen, Germany. *Informed consent*: All participants gave written informed consent before enrolment. *Registry and the registration no. of the study/trial*: N/A. *Animal studies*: N/A. *Conflict of interest*: The authors declare that they have no competing of interests.

## AUTHOR CONTRIBUTION

All listed authors have significantly contributed to this work. CP, KK, and FCM conceived and designed research. CW, CP, TR, TF, and FCM conducted the experiment. CW analyzed the data and wrote the manuscript with CP and KK. All authors read and approved the final manuscript.
